# Effects of Oat Bran on Nutrient Digestibility, Intestinal Microbiota, and Inflammatory Responses in the Hindgut of Growing Pigs

**DOI:** 10.3390/ijms19082407

**Published:** 2018-08-15

**Authors:** Beibei He, Yu Bai, Lili Jiang, Wei Wang, Tiantian Li, Ping Liu, Shiyu Tao, Jiangchao Zhao, Dandan Han, Junjun Wang

**Affiliations:** 1 Beijing Advanced Innovation Center for Food Nutrition and Human Health, College of Animal Science and Technology, China Agricultural University, Beijing 100193, China; beibei_he@hotmail.com (B.H.); vei.wang@hotmail.com (W.W.); caultt@hotmail.com (T.L.); 2 State Key Laboratory of Animal Nutrition, College of Animal Science and Technology, China Agricultural University, Beijing 100193, China; yubaijlucau@163.com (Y.B.); 15738843897@163.com (L.J.); Pingliu2010@163.com (P.L.); 18761867726@163.com (S.T.); handandan2012@163.com (D.H.); 3 Department of Animal Science, University of Arkansas, Fayetteville, AR 72701, USA; jzhao77@uark.edu

**Keywords:** oat bran, nutrient digestibility, intestinal microbiota, inflammatory responses

## Abstract

Oat bran has drawn great attention within human research for its potential role in improving gut health. However, research regarding the impact of oat bran on nutrient utilization and intestinal functions in pigs is limited. The purpose of this study was to investigate the effects of oat bran on nutrient digestibility, intestinal microbiota, and inflammatory responses in the hindgut of growing pigs. Twenty-six growing pigs were fed either a basal diet (CON) or a basal diet supplemented with 10% oat bran (OB) within a 28 day feeding trial. Results showed that digestibility of dietary gross energy, dry matter, organic matter, and crude protein were lower in the OB group compared to the CON group on day 14, but no differences were observed between the two groups on day 28. In the colon, the relative abundance of operational taxonomic units (OTUs) associated with *Prevotella*, *Butyricicoccus*, and *Catenibacterium* were higher, while those associated with *Coprococcus* and *Desulfovibrio* were lower in the OB group compared to the CON group. Oat bran decreased mRNA expression of caecal interleukin-8 (IL-8), as well as colonic IL-8, nuclear factor-κB (NF-κB), and tumor necrosis factor-α (TNF-α) of the pigs. In summary, oat bran treatment for 28 day did not affect dietary nutrient digestibility, but promoted the growth of cellulolytic bacteria and ameliorated inflammatory reactions in the hindgut of growing pigs.

## 1. Introduction

Dietary fiber (DF) including non-starch polysaccharides, lignin, non-digestible oligosaccharides, and resistant starch is not hydrolyzed by endogenous enzymes in the small intestine and becomes available for bacterial fermentation in the large intestine [1]. For monogastric animals such as pigs, high fiber content in the diet was usually associated with decreased nutrient and energy digestibility [2]. However, the negative impact of fiber on the nutrient utilization of pigs varied between different fiber sources and different fiber properties [3,4]. Recently, there has been increased interest in the application of DF in pig nutrition, not only for economic reasons, but also for its potential roles in promoting gut health and improving the innate immune defense. DF fermentation in the hindgut of pigs results in the production of short chain fatty acids (SCFAs), which may be utilized by the intestinal cells as energy sources or available for the growth of beneficial bacteria [5]. In addition, various types of DF have been shown to enhance the intestinal barrier functions and ameliorate inflammatory responses, thereby promoting overall gut health in pigs [6,7].

Oat bran is one of the major byproducts in the processing of husked oat, which contain relatively high levels of protein, minerals, vitamins, and soluble β-glucan [8]. Previous studies have shown that oat bran, supplemented with oil, may be useful in the diet of growing pigs [9,10]. It was demonstrated that oat bran can increase energy digestibility and fiber utilization of gestating sows better than wheat straw and sugar beet pulp [3]. The DF components of oat bran that escape enzymatic digestion in the small intestine are almost entirely fermented by bacteria in the large intestine due to their water-soluble and fermentable properties [11], and produce almost twice as many SCFAs as wheat bran [9]. Oat bran was found to stimulate the growth of beneficial bacteria and exert a positive response on improving gut health [12]. What’s more, a few studies reported that oat bran may reduce the oxidative stress and inflammatory responses [13,14]. Thus, oat bran becomes an important consideration as an alternative feed ingredient in swine production. However, continued investigations are needed to clarify the beneficial role of oat bran regarding the gut health of pigs.

The objective of this study was to test the hypothesis that the addition of oat bran to pig diet could affect nutrient digestibility, intestinal microbiota composition, and fermentation profiles, as well as inflammatory responses in the hindgut of growing pigs.

## 2. Results

### 2.1. The Growth Performance of Growing Pigs Fed either Control or Oat Bran Diet

The growth performances of growing pigs fed the control diet (CON) compared to the oat bran diet (OB) during the 28 day experiment period are shown in Table 1. The average daily gain (ADG), average daily feed intake (ADFI), and feed conversion ratio (FCR) were virtually the same (*p* > 0.05) between the CON and OB groups.

### 2.2. Nutrient Digestibility of Growing Pigs Fed either Control or Oat Bran Diet

The apparent total tract digestibility (ATTD) of gross energy (GE), dry matter (DM), organic matter (OM), crude protein (CP), neutral detergent fiber (NDF), and acid detergent fiber (ADF) in growing pigs fed the CON or the OB diet were determined on day 14 and day 28, respectively (Table 2). Compared to the CON group, ATTD of GE, DM, OM, and CP decreased (*p* < 0.05) in the OB group on day 14, while ATTD of NDF and ADF were the same between the two groups. On day 28, the ATTD of GE, DM, OM, CP, and ADF were the same between the two groups, while the ATTD of NDF was higher (*p* < 0.05) in the OB group compared to the CON group (Table 2).

### 2.3. Sequencing and Taxonomic Composition of the Caecal and Colonic Digesta Microbiota of the CON and OB Groups

Using high-throughput 16S rRNA gene sequencing, we obtained averages of 37,815 and 34,131 high-quality sequences from the caecal and colonic digesta samples, respectively, which were further clustered into averages of 415 and 482 operational taxon units (OTUs) at the 97% sequence similarity level. The sequences were deposited into the NCBI Sequence Read Archive database (accession number: SRP150170).

**Figure 1:**
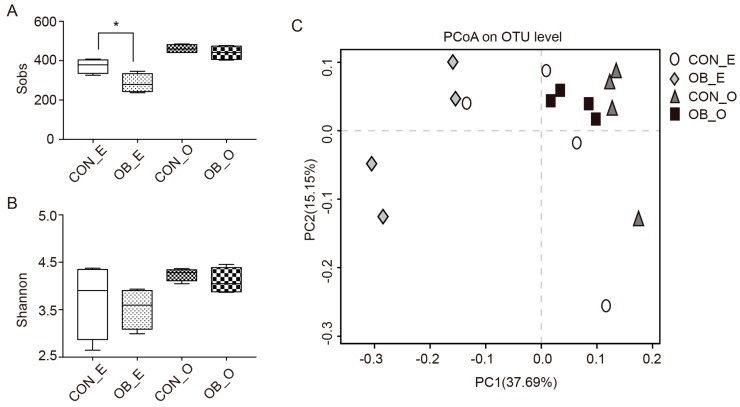
The microbiota α- and β-diversity of the caecal (E) and colonic (O) digesta from growing pigs fed either control (CON) or oat bran diet (OB). Each symbol represents a different group. (**A**) The Sobs index of the caecal and colonic digesta microbiota of the CON and OB groups; (**B**) the Shannon index of the caecal and colonic digesta microbiota of the CON and OB groups; (**C**) the principal coordinates analysis (PCoA) plots of the microbial communities at operational taxon unit (OUT) level. Values are mean ± SEM, *n* = 4. * Compared with the control *p* < 0.05.

Richness and diversity of bacterial communities were measured by α-diversity indices. The Sobs index (i.e., observed number of OTUs) was lower (*p* < 0.05) in the caecal digesta of the OB group compared to the CON group, but was similar in the colonic digesta bacteria between the two groups (Figure 1A). The Shannon indices were the same (*p* > 0.05) between the two groups regarding the caecal and colonic digesta (Figure 1B). Principal coordinates analysis (PCoA) plot based on the unweighted unifrac showed that there were significantly differences (*p* < 0.05) in the microbiota membership between the caecal and colonic digesta, but no differences in microbiota membership were found between the CON and OB groups (Figure 1C).

**Figure 2:**
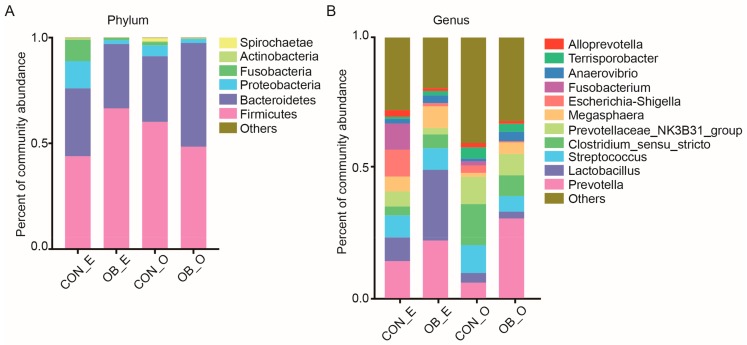
Microbiota composition in the caecal (E) and colonic (O) digesta from growing pigs fed either control (CON) or oat bran diet (OB). (**A**) Microbiota composition in the caecal and colonic digesta at the phylum level; (**B**) microbiota composition in the caecal and colonic digesta at the genus level. The results were presented as mean percentage of different bacteria, *n* = 4.

The microbiota composition was reported both at the phylum and genus levels. At the phylum level, the microbiota of the caecal and colonic digesta were dominated by Firmicutes and Bacteroidetes in the CON and OB groups (Figure 2A). At the genus level, OTUs associated with *Lactobacillus* (18.1%), *Prevotella* (18.1%), and *Streptococcus* (8.4%) were predominant in the caecal digesta of the CON and OB groups, while OTUs associated with *Prevotella* (18.2%), *Clostridium_sensu_stricto* (11.9%), and *Prevotellaceae_NK3B31_group* (9.4%) were predominant in the colonic digesta of both groups (Figure 2B).

### 2.4. Different Abundant Genera in the Caecal and Colonic Digesta of the CON and OB groups

To identify changes in relative abundance of genera between the two groups, we performed linear discriminant analysis effect size (LEfSe), which not only detects statistical difference but also emphasizes biological consistence. In the caecal digesta, the relative abundance of *Catenibacterium* was higher (*p* < 0.05), while the relative abundance of *Peptococcus* was lower (*p* < 0.05) in the OB group compared to the CON group (Table 3). In the colonic digesta, the relative abundance of OTUs belonging to *Prevotella*, *Butyricicoccus*, *and Catenibacterium* were higher (*p* < 0.05), while the relative abundance of OTUs classified as *Coprococcus* and *Desulfovibrio* were lower (*p* < 0.05) in the OB group compared to the CON group (Table 3).

### 2.5. The Concentration of SCFAs in the Caecal and Colonic Digesta of the CON and OB Groups

The concentrations of acetic acid, propionic acid, and butyric acid were examined in the caecal and colonic digesta of the CON and OB groups (Table 4). The results showed that the concentrations of SCFAs in the caecal digesta of both groups were similar. In the colonic digesta, the concentration of propionic acid was higher (*p* < 0.05) in the OB group than that of the CON group, while other levels of detected SCFAs were comparable between the two groups (Table 4).

### 2.6. Gene Expression in the Caecum and Colon of the CON and OB Groups

**Figure 3:**
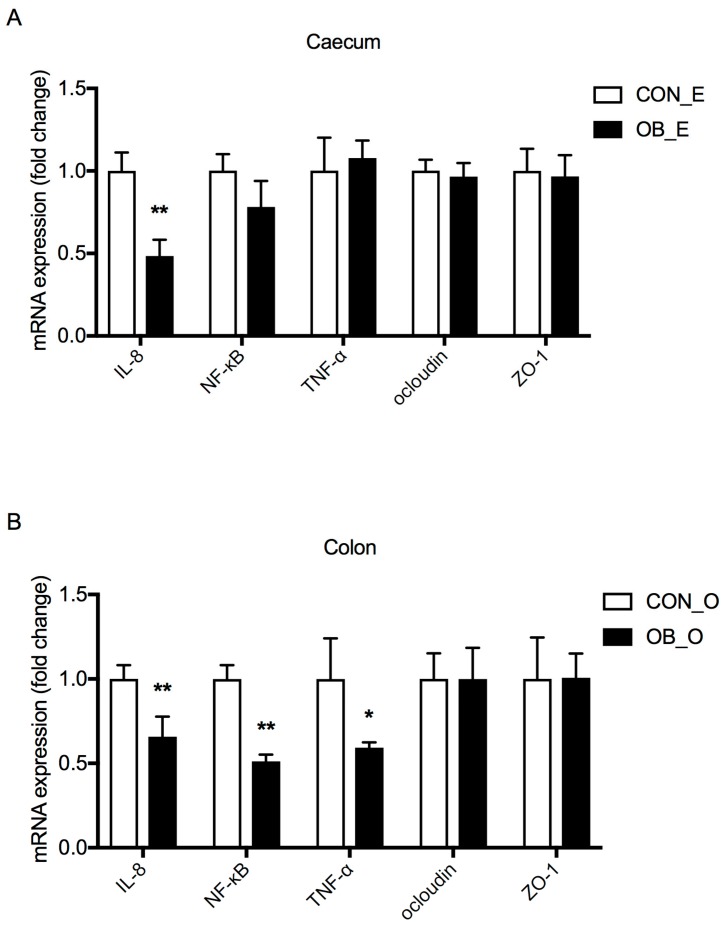
Gene expression in the caecum (E) and colon (O) of growing pigs fed either control (CON) or oat bran diet (OB). (**A**) Intestinal gene expression in the caecum; (**B**) intestinal gene expression in the colon. Values are mean ± SEM, *n* = 4. Interleukin-8 (IL-8); nuclear factor-κB (NF-κB); tumor necrosis factor-α (TNF-α); zonula occludens-1 (ZO-1). * Compared with the control *p* < 0.05. ** Compared with the control *p* < 0.01.

The mRNA expressions of genes related to energy metabolism, inflammation, and barrier functions in the caecum and colon of pigs were determined using the method of reverse transcription quantitative real-time PCR (RT-qPCR). The mRNA expressions of interleukin-8 (IL-8) was lower (*p* < 0.05) in the caecum of the OB group compared to the CON group (Figure 3A). Dietary inclusion of oat bran also decreased (*p* < 0.05) the mRNA expression of IL-8, nuclear factor-κB (NF-κB), and tumor necrosis factor-α (TNF-α) in the colon (Figure 3B). However, there were no differences in the mRNA expression of occludin and zonula occludens-1 (ZO-1) between the two groups (Figure 3).

## 3. Discussion

In past decades, the rapid development of swine production was accompanied by growing concerns about the economic pressure of feed cost and antibiotic resistance [15]. Inclusion of DF in the diet was thought to be an effective way to reduce feed cost and improve the gut health of pigs. Oat bran was considered an important alternative feed ingredient in swine production. However, there is still a need for more research to understand the functional roles of oat bran in the pig gastrointestinal tract and its application in swine production. In this study, pigs were fed either a conventional corn-soybean meal diet (basal diet) or a basal diet supplemented with 10% oat bran, to determine the effect of oat bran on growth performance, nutrient digestibility, microbiota composition, and fermentation profiles, as well as the inflammatory responses in the hindgut of growing pigs.

Dietary administration of oat bran did not affect ADG or ADFI of the pigs during the experimental period compared to the basal diet treatment (Table 1). The digestibility of dietary GE, DM, OM, and CP was lower in the OB group on day 14, compared to the CON group (Table 2). This was consistent with other studies that reported the physico-chemical properties of oat bran, such as viscosity and water solubility, which may increase digesta viscosity and limit the interaction between nutrients and enzymes in the small intestine [16], thereby reducing nutrient digestion and absorption. However, nutrient digestibility was not affected by the addition of oat bran after the 28 day treatment (Table 2). DF fermentation in the large intestine can result in the production of SCFAs, and the energy produced from these metabolically important molecules may contribute up to 15% of the energy maintenance requirements of growing pigs [17]. In our results, the concentration of propionic acid was higher in the colonic digesta of the OB group compared to the CON group (Table 4), which may partly help improve the nutrient digestibility of the OB group. In addition, previous studies showed that intestinal bacteria will adapt and ferment complex carbohydrates more efficiently [18,19]. Although the nutrient digestibility was lower in the OB group compared to the CON group on day 14, the enhanced fiber fermentation and increased SCFAs production during subsequent weeks may lead to a similar nutrient digestibility in the OB group on day 28, compared to the CON group.

Fiber components in the diet are important factors that influence the intestinal bacteria in swine [5,12]. Using 16S rRNA sequencing, we determined the microbiota composition in the caecal and colonic digesta of the CON and OB groups. The Sobs index was lower in the caecal digesta of the OB group compared to the CON group, while the Shannon index in the colonic digesta was the same between the two groups (Figure 1). Consistent with previous studies, the microbiota of the caecal and colonic digesta from both groups were dominated by Firmicutes and Bacteroidetes (Figure 2) [20]. At the genus level, the abundance of *Peptococcus* was lower in the caecal digesta of the OB group, and *Catenibacterium* was higher in the caecal digesta of the OB group compared to the CON group (Table 3). *Peptococcus* was frequently isolated from piggery wastes [21], while *Catenibacterium* was once discovered to have a significant increased abundance in pigs infected with *Salmonella enterica* [22,23]. Information about these two genera, for the most part, is currently lacking and thus there exists a great need for further research of these bacteria. As to the colonic digesta, the relative abundance of *Prevotella*, *Butyricicoccus*, and *Catenibacterium* were higher in the OB group compared to the CON group. The genus *Prevotella* was found to be positively correlated with the production of SCFAs and the metabolism of amino acids, energy, cofactors, and vitamins in the host [24]. Specifically, the presence of *Prevotella* decreased in pigs suffering from post-weaning diarrhea [25,26]. *Butyricicoccus*, a butyrate-producing genera belonging to the family Ruminococcaceae, had a higher abundance in fecal samples from pigs fed whole grain barley and oat diet, compared to pigs fed the extruded cereal diet [27]. Consistently, the concentration of propionic acid in the colonic digesta of the OB group remained higher than that of the CON group. Therefore, the colonic digesta of pigs fed an oat bran diet may be predominated with cellulolytic bacteria, such as *Prevotella* and *Butyricicoccus*, resulting in a higher production of SCFAs, which in turn provides a more sustained homeostatic balance leading to a heathier gut. Two other genera, *Coprococcus* and *Desulfovibrio*, were discovered to have a lower abundance in the colonic digesta of the OB group compared to the CON group (Table 4). Previous studies reported that the abundance of genus *Coprococcus* was significantly higher in the hindgut of pigs fed a diet containing a high level of resistant starch [28], and several species of the genus *Coprococcus* were associated with the production of butyric acid [29]. The genus *Desulfovibrio* was discovered to have a higher abundance in pigs fed a pea fiber diet with a possible connection to fiber degradation [30]. Although *Coprococcus* and *Desulfovibrio* may participate in fiber digestion, the negligible portion of these bacteria in the colonic digesta of pigs may weaken their contributions, compared to other cellulolytic bacteria, such as *Prevotella*. In addition, several *Desulfovibrio* species were considered as significant features in identifying dysentery and intestinal dysbiosis [31,32], which was lower in the colonic digesta of the OB group.

The presence of DF in the hindgut affects intestinal microbial environment, leading to a possible connection to changes in intestinal functions. Oat bran and its fiber components have been well studied for their beneficial role in alleviating oxidative stress and inflammatory responses in humans [33,34]. In this study, the mRNA expression of IL-8 was lower in the caecum of the OB group compared to the CON group, while the mRNA expressions of IL-8, NF-κB, and TNF-α were lower in the colon of the OB group (Figure 3). Intestinal pro-inflammatory cytokines, such as IL-8 and TNF-α, have been shown to increase intestinal permeability through the dysregulation of tight junction proteins [35,36]. NF-κB is an important transcription factor involved in the regulation of inflammation and immune responses [37]. Previous studies have shown that the phenolic compounds present in oat bran have a beneficial effect on the oxidative stability of pig meat [13], and oat bran intake effectively reduced oxidative stress induced by a high-fat diet in pigs [14]. Therefore, decreased mRNA expressions of the inflammation factors in the OB group confirmed the functional roles of oat bran in alleviating inflammatory responses in the hindgut of growing pigs, which may greatly contribute to improved gut health. Previous studies have also concluded that DF improved the intestinal barrier functions of the ileum and colon in weaned piglets, a result which was probably mediated by changes in the microbiota composition [6]. For example, the mRNA expression of occludin, ZO-1, ZO-2, and cingulin were upregulated by *Lactobacillus* [38], and *Escherichia coli* could disassemble the tight junction structure of epithelial cells [39]. However, in this study, the addition of oat bran did not affect the mRNA expression of ZO-1 and occludin in the caecum or colon (Figure 3). A possible reason for this may be that the varied digestible and fermentable ability of different DF sources could have different effects on intestinal barrier functions.

In summary, oat bran inclusion at 10% in the diet had no effect on growth performance and nutrient digestibility of pigs on day 28 of the trial. Oat bran enriched the abundance of *Prevotella*, *Butyricicoccus,* and *Catenibacterium* in the colonic digesta. Increasing the relative abundance of these bacteria may enhance the fermentation of fiber to produce SCFAs, thereby improving gut health and nutrient utilization. In addition, oat bran decreased mRNA expression of IL-8 in the caecum and reduced IL-8, NF-κB, and TNF-α gene levels in the colon. Such results emphasize the functional roles of oat bran on ameliorating inflammatory responses in the hindgut of growing pigs.

## 4. Materials and Methods

### 4.1. Animals and Design

The treatment, housing, husbandry, and slaughtering conditions used in this study was approved by the Institutional Animal Care and Use Committee of China Agricultural University (No. CAU20170403-1, 03 April 2017). Twenty-six crossbred (Duroc × Landrace × Large White) barrows with the initial body weight (BW) of 30.5 ± 2.6 kg were selected and randomly divided into two pens equipped with automatic feeding system (HAMOER Technology Co., Ltd., Beijing, China), which recorded pig feed intake and body weight individually by recognizing the electronic ear mark every time they eat. The temperature of the pig house was maintained at 22 °C and the humidity at 65~75%. Feed and water were provided ad libitum, all pigs were healthy and none received antibiotic treatment during the experimental period. Pigs in the two pens were fed either a control diet based on corn-soybean meal, or a control diet supplemented with 10% oat bran (Table 5). The oat bran was separated from naked oat residual and after polishing and milling was made to pass through a 1 mm sieve before adding to the OB formula. Chromic oxide was added as a marker at a concentration of 0.3%. Diets were formulated according to the nutritional requirements of the National Research Council (NRC, 1998, the United States) for pigs weighing 20 to 50 kg.

### 4.2. Sample Collection

Diet samples were collected for chemical analysis. Fecal samples were collected on day 14 and day 28 of the trials. Diets and fecal samples were pooled by replicate and dried at 65 °C for 72 h. Samples were ground to pass through a 40-mesh screen and stored at −20 °C until analysis for nutrient digestibility. On day 28, four pigs in each group were selected based on their ADFI and ADG. After slaughtering via electrical stunning followed by exsanguination, these pigs were bled and their abdomen was opened immediately. The caecum and mid colon was excised for tissue and digesta collection. All samples were immediately immersed in liquid nitrogen and subsequently stored at −80 °C for future analysis.

### 4.3. Chemical Analysis of Feed and Fecal Samples

Diet and fecal samples were analyzed for GE, DM, OM, and CP according to standard Association of Official Analytical Chemists (AOAC) methods. NDF and ADF were measured according to the Van Soest method [40]. The chromium content was measured using a spectrophotometer (Hitachi Z-5000 Absorption spectrophotometer, Hitachi High-Technologies Company, Tokyo, Japan) based on the report by Williams et al. [41].

### 4.4. DNA extraction, PCR amplification, and Illumina Sequencing for Intestinal Microbiota

Total bacterial DNA was extracted from the caecal and colonic digesta using the QIAamp Fast DNA stool mini kit (51604, Qiagen, Hilden, Germany). Barcoded amplicons from the V3-V4 region of 16S rRNA genes were generated by PCR amplification. After purification, amplicons were pooled and paired-end sequenced on the MiSeq Illumina platform (Illumina Inc., San Diego, CA, USA). The raw reads were deposited into the NCBI Sequence Read Archive database (accession number:SRP150170). Raw sequences were demultiplexed and quality filtered using QIIME 1.17. Only sequences with an overlap longer than 10 base pair reads and without any mismatch were assembled according to their overlap sequence. OTUs were clustered with 97% similarity cutoff using UPARSE (vsesion 7.1 http://drive5.com/uparse/) and chimeric sequences were removed. The Ribosomal Database Project (RDP) Classifier (http://rdp. cme.msu.edu/) was used to analyze the phylogenetic affiliation of each 16S rRNA gene sequence with confidence greater than 70%. PCoA analysis based on unweighted UniFrac distance metrics was conducted according to the matrix of distance using JMP software of Statistical Analysis System (SAS) (version 8.0.2, SAS Institute, Cary, NC, USA) [42].

### 4.5. Analysis of the SCFAs in the Caecal and Colonic Digesta

The concentrations of acetic acid, propionic acid, and butyric acid in the caecal and colonic digesta were determined using a high performance ion chromatography system (DIONEX ICS-3000, Thermo Fisher, Waltham, MA, USA). Digesta samples were weighed (~0.5 g) and dissolved in 8 mL ultrapure water. After ultrasound for 30 min, digesta samples were centrifuged at 3000× *g* for 10 min. The suspension was then diluted (1:50) and filtered through a 0.22 μm membrane before injection into an AG11 guard column (250 mm × 4 mm) and an AG11 guard column using KOH for isocratic elution. The injection volume was 25 μL and the flow rate was 1.0 mL/min [43].

### 4.6. Real-time Quantitative PCR (RT-qPCR) Analysis

Total RNA of caecum and colon tissues were extracted using Trizol reagent (RN0101, Aidlab Biotechnologies Co., Ltd., Beijing, China) following the manufacturer’s instructions. The RNA integrity and purity was checked using a Thermo Scientific NanoDrop spectrophotometer (NanoDrop 1000, Thermo Fisher, Waltham, MA, USA). RT-qPCR was performed using Synergy Brands (SYBR) Premix Ex Taq II (RR420A, TaKaRa, Shanghai, China) and the Roche LightCycler96 fluorescent quantitative PCR (LightCycler96, Roche, Basel, Sweden) according to the manufacturer’s instructions. The primers used in this study are listed in Table 6. The mRNA level of β-actin was used as the internal control. The 2^−ΔΔC*T*^ method was used to determine the fold changes in mRNA levels of each sample, of which included a control reference sample.

### 4.7. Statistical Analysis

Data analyses of growth performance, nutrient digestibility, SCFA concentrations, and gene expression were performed with Statistical Product and Service Solutions (SPSS) (version 21.0, SPSS Inc., Chicago, IL, USA) by independent sample *t* test, with the results presented as mean values ± SEM. Probability values ≤0.05 were considered significant.

## Figures and Tables

**Table 1:** The growth performance of pigs fed either control or oat bran diet during the 28 day experimental period ^1^.

Item	CON	OB	*p*-Value
ADG (kg/d)	0.61 ± 0.03	0.67 ± 0.03	0.12
ADFI (kg/d)	1.33 ± 0.05	1.35 ± 0.04	0.67
FCR	2.22 ± 0.11	2.06 ± 0.10	0.29

^1^ Values are mean ± SEM, *n* = 13. CON, control group; OB, oat bran group; ADG, average daily gain; ADFI, average daily feed intake; FCR, feed conversion ratio.

**Table 2:** Nutrient digestibility of pigs fed either control or oat bran diet on day 14 and day 28 of the experimental period ^1^.

Item%	CON	OB	*p*-Value
day 14			
GE	91.03 ± 0.32 ^a^	89.26 ± 0.11 ^b^	<0.01
DM	91.31 ± 0.30 ^a^	89.47 ± 0.12 ^b^	<0.01
OM	93.07 ± 0.23 ^a^	91.43 ± 0.08 ^b^	<0.01
CP	89.59 ± 0.27 ^a^	86.82 ± 0.58 ^b^	<0.01
NDF	77.78 ± 1.30	80.87 ± 1.62	0.19
ADF	78.44 ± 2.48	75.67 ± 1.86	0.41
day 28			
GE	88.76 ± 1.03	88.42 ± 0.67	0.79
DM	89.14 ± 0.90	88.59 ± 0.61	0.63
OM	92.84 ± 0.96	91.05 ± 0.52	0.15
CP	86.25 ± 1.86	88.04 ± 0.56	0.39
NDF	68.97 ± 2.85 ^b^	78.81 ± 1.89 ^a^	0.03
ADF	70.84 ± 3.54	70.16 ± 2.83	0.89

^1^ Values are mean ± SEM, *n* = 4. CON, control group; OB, oat bran group; GE, gross energy; DM, dry matter; OM, organic matter; CP, crude protein; NDF, neutral detergent fiber; ADF, acid detergent fiber. ^a, b^ Statistically significant differences within rows are annotated with different letters *p* < 0.05.

**Table 3:** The different abundant genera in the caecal and colonic digesta of pigs fed either control or oat bran diet ^1^.

Item%	CON	OB	*p-*Value
Caecal Digesta			
*Peptococcus*	0.45 ± 0.16 ^a^	0.07 ± 0.02 ^b^	0.04
*Catenibacterium*	0.02 ± 0.01 ^b^	0.37 ± 0.06 ^a^	<0.01
Colonic Digesta			
*Prevotella*	7.89 ± 0.03 ^b^	32.91 ± 0.06 ^a^	<0.01
*Coprococcus*	1.37 ± 0.18 ^a^	0.80 ± 0.09 ^b^	0.03
*Desulfovibrio*	0.39 ± 0.10 ^a^	0.11 ± 0.01 ^b^	0.03
*Butyricicoccus*	0.10 ± 0.04 ^b^	0.33 ± 0.06 ^a^	0.02
*Catenibacterium*	0.01 ± 0.01 ^b^	0.13 ± 0.02 ^a^	<0.01

^1^ Values are mean ± SEM, *n* = 4. CON, control group; OB, oat bran group. ^a, b^ Statistically significant differences within rows are annotated with different letters *p* < 0.05.

**Table 4:** Short chain fatty acid (SCFA) concentrations in the caecal and colonic digesta of pigs fed either control or oat bran diet ^1^.

Item, mg/g	CON	OB	*p-*Value
Caecal Digesta			
Acetic acid	0.99 ± 0.10	0.93 ± 0.02	0.59
Propionic acid	0.62 ± 0.02	0.69 ± 0.05	0.25
Butyric acid	0.16 ± 0.00	0.16 ± 0.02	0.94
Colonic Digesta			
Acetic acid	0.94 ± 0.10	1.19 ± 0.04	0.07
Propionic acid	0.56 ± 0.08 ^b^	0.89 ± 0.04 ^a^	< 0.01
Butyric acid	0.23 ± 0.03	0.25 ± 0.04	0.55

^1^ Values are mean ± SEM, *n* = 4. CON, control group; OB, oat bran group. ^a, b^ Statistically significant differences within rows are annotated with different letters *p* < 0.05.

**Table 5:** Composition and nutrient analysis of experimental diet ^1^ (as-fed basis).

Item	CON	OB
Ingredients%		
Corn	74.39	65.75
Soybean meal	22.40	20.00
Oat bran	0.00	10.00
Dicalcium phosphate	0.62	0.62
Limestone	0.76	0.78
Salt	0.35	0.35
Vitamine/mineral premix ^2^	0.50	0.50
Soybean oil	0.00	1.00
L-Lysine-HCl	0.40	0.45
DL-Methionine	0.08	0.05
L-Threonine	0.12	0.12
L-Tryptophan	0.02	0.02
L-Valine	0.06	0.06
Cr_2_O_3_	0.30	0.30
Nutrient analysis ^3^		
Crude protein, %	16.49	16.33
Calcium, %	0.57	0.50
Phosphorus, %	0.45	0.49
Metabolic energy, MJ/kg	13.74	13.57
Total Lysine, %	0.96	1.03
TDF, %	13.41	14.96
IDF, %	11.56	12.17
SDF, %	1.86	2.80

^1^ CON, control diet; OB, oat bran diet; TDF, total dietary fiber; IDF, insoluble dietary fiber; SDF, soluble dietary fiber. ^2^ Supplied per kilogram of diet: vitamin A, 6.0 KIU; vitamin D3, 2.4 KIU; vitamin E, 21.6 IU; vitamin K, 2.0 mg; thiamine 1.0 mg; riboflavin, 5.2 mg; pyridoxine, 2.0 mg; vitamin B12, 0.01 mg; D-pantothenic acid, 11.2 mg; niacin, 22 mg; biotin, 40 μg; folic acid, 0.4 mg; Fe, 120 mg; Zn, 120 mg; Mn, 40.0 mg; Cu, 80 mg; I, 400 μg; Se, 240 μg; Ca 8.0 g; P, 0.4 g. ^3^ The nutrient levels were analyzed values. Metabolic energy values were calculated.

**Table 6:** Primers for reverse transcription quantitative real-time PCR (RT-qPCR) used in this study.

Gene	Sequences	Accession No.
*Zonula occludens-1 (ZO-1)*	Forward: ATCTCGGAAAAGTGCCAGGA	XM_021098856.1
	Reverse: CCTTCCCCTCAGAAACCCAT	
*Occludin*	Forward: CAGCCTCATTACAGCAGCAG	NM_001163647.2
	Reverse: AGCTCTTGTACTCCTGCAGG	
*Interleukin-8 (IL-8)*	Forward: TCCAAACTGGCTGTTGCCTT	NM_213867.1
	Reverse: TCCAAACTGGCTGTTGCCTT	
*Nuclear factor-κB (NF-κB)*	Forward: GGCTATAACTCGCTTGGTGACAGG	NM_001048232.1
	Reverse: CCGCAATGGAGGAGAAGTCTTCG	
*Tumor necrosis factor-α (TNF-α)*	Forward: GCACTGAGAGCATGATCCGAGAC	NM_214022.1
	Reverse: CGACCAGGAGGAAGGAGAAGAGG	
*β-actin*	Forward: TCTGGCACCACACCTTCTACA	XM_021086047.1
	Reverse: ATCTGGGTCATCTTCTCACGG	

## Abbreviations

IL-8	Lnterleukin-8
NF-κB	Nuclear factor-κB
TNF-α	Tumor necrosis factor-α
ZO-1	Zonula occludens-1
SCFA	Short chain fatty acid
DF	Dietary fiber
IDF	Insoluble dietary fiber
SDF	Soluble dietary fiber
ADG	Average daily gain
ADFI	Average daily feed intake
FCR	Feed conversion ratio
ATTD	Apparent total tract digestibility
GE	Gross energy
DM	Dry matter
OM	Organic matter
CP	Crude protein
NDF	Neutral detergent fiber
ADF	Acid detergent fiber
OTU	Operational taxon unit
PCoA	Principal coordinates analysis
LEfSe	Linear discriminant analysis effect size
LDA	Linear discriminant analysis
